# Efficacy of renal denervation as an adjunct to pulmonary vein isolation for atrial fibrillation treatment: a systematic review and meta-analysis

**DOI:** 10.1093/ehjopen/oeae065

**Published:** 2024-08-05

**Authors:** Karish Thavabalan, Majed Sheikh, YuZhi Phuah, Sanjay K Rajput, Noor Fatima, Aman Sutaria, Jonathan J H Bray, Mahmood Ahmad, Hannah Glatzel, Reubeen Ahmad, Lily Snell, Niraj S Kumar, Carmen-Lucía García-Pérez, Luciano Candilio, Rui Providencia

**Affiliations:** University College London Medical School, 74 Huntley St, London WC1E 6DE, UK; Department of Cardiology, Royal Free Hospital, London, UK; University College London Medical School, 74 Huntley St, London WC1E 6DE, UK; University College London Medical School, 74 Huntley St, London WC1E 6DE, UK; University College London Medical School, 74 Huntley St, London WC1E 6DE, UK; University College London Medical School, 74 Huntley St, London WC1E 6DE, UK; University College London Medical School, 74 Huntley St, London WC1E 6DE, UK; Oxford Heart Centre, Oxford University Hospitals Trust, John Radcliffe Hospital, Headley Way, Headington, Oxford, UK; Tahir Heart Institute, Rabwah, Pakistan; Stoke Mandeville Hospital, Aylesbury, UK; Brighton and Sussex Medical School, Brighton, UK; University College London Medical School, 74 Huntley St, London WC1E 6DE, UK; University College London Medical School, 74 Huntley St, London WC1E 6DE, UK; National Medical Research Association, UK; University College London Medical School, 74 Huntley St, London WC1E 6DE, UK; The Hatter Cardiovascular Institute, University College London, London, UK; Institute of Health Informatics Research, 222 Euston Road, NW1 2DA London, UK; St Bartholomew's Hospital, Barts Heart Centre, Barts Health National Health Service Trust, West Smithfield, EC1A 7BE London, UK

**Keywords:** Atrial fibrillation, Renal denervation, Hypertension

## Abstract

**Aims:**

Catheter ablation, consisting of pulmonary vein isolation (PVI), is the most effective treatment modality for the management of symptomatic patients with atrial fibrillation (AF). Unfortunately, this procedure has a considerable relapse rate, ranging from 15 to 50% depending on AF type and other patient factors. Hypertension (HTN) is associated with a higher risk of developing AF and can also be managed with a catheter-based procedure—renal denervation (RDN). This meta-analysis aimed to compare the effect of PVI with and without RDN in hypertensive patients with AF.

**Methods and results:**

OVID MEDLINE and Embase were searched on 1 February 2023 and trials that reported the effects of RDN on AF recurrence in hypertensive patients were included. A total of 637 patients across 8 randomised controlled trials were included. The results from the pooled analysis showed that when compared with PVI alone, RDN added to PVI: (1) Lowered AF recurrence [RR 0.67 (0.53, 0.85), *P* = 0.001, *I*^2^ = 23%, NNT = 5.9 patients]; (2) Reduced both systolic blood pressure and diastolic blood pressure, with medium effect size, as reflected by standardised mean differences of 0.5 (*P* = 0.02, *I*^2^ = 80%) and 0.43 (*P* = 0.006, *I*^2^ = 60%), respectively; and (3) was not associated with a decrease in estimated glomerular filtration rate (+7.19 mL/min/1.73 m^2^, *P* = 0.15, *I*^2^ = 89%).

**Conclusion:**

Adding RDN to PVI in patients with AF and resistant HTN was associated with a reduction of blood pressure levels and AF recurrence. Consideration to RDN should be given as an adjunctive treatment for patients with AF and resistant HTN.

## Graphical Abstract

**Graphical abstract:**
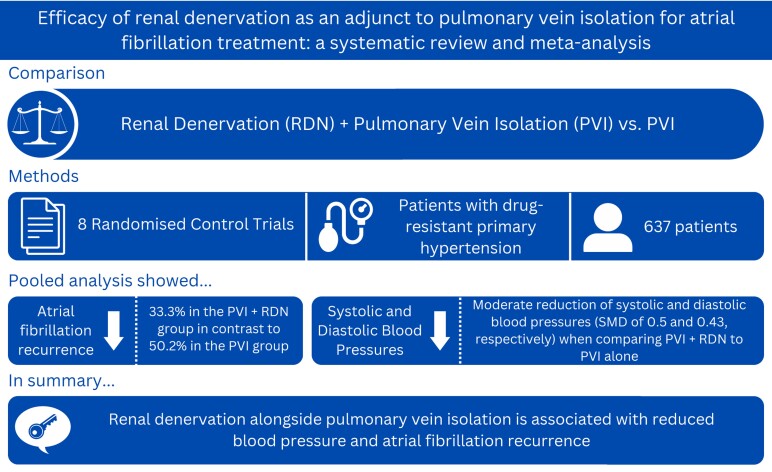

## Introduction

Atrial fibrillation (AF) is the commonest cardiac arrhythmia, with its global prevalence expected to rise dramatically over the next 40 years. By 2060, it is estimated that 17.9 million patients will be diagnosed with AF in the European Union alone, over double the current figure.^1^ AF is also associated with an increased risk of cardiovascular and cerebrovascular mortality as well as significant costs for inpatient stays, outpatient and emergency department attendances, diagnostic tests and treatment.^1^

Hypertension (HTN) is one of the most prevalent risk factors involved in the pathogenesis of AF, with data from the ARIC study suggesting that more than one-fifth of AF cases are attributable to HTN.^2^ Hence, haemodynamic control has been a focus of research in the prevention and treatment of drug-refractory AF. Renal denervation (RDN) has been shown to reduce blood pressure in patients with uncontrolled or resistant HTN.^3^

The pulmonary veins contain triggers that can cause AF, and pulmonary vein isolation (PVI) is a therapeutic option for this arrhythmia.^4^ This catheter-based procedure involves electrically isolating the four pulmonary veins from the left atrial myocardium.^5^ However, despite PVI being a successful means of reducing the burden of AF, around 15–50% of patients will have AF recurrence in the first year, necessitating reintervention.^6,7^

It is therefore plausible that RDN added to PVI in patients with AF and HTN, may lead to a better blood pressure control, hence reducing further the risk of AF recurrence. This analysis aimed to compare the effect of PVI with and without RDN in hypertensive patients with AF. Primary outcomes of interest were AF recurrence and blood pressure control. Additionally, the overall safety of the procedures and estimated glomerular filtration rate (eGFR) were examined as secondary outcomes.

## Methods

### Literature search and screening

The research question was framed using the PICO structure: Patients—Patients with AF and HTN; Intervention—PVI + RDN; Control—PVI; Outcome—Procedural efficacy and safety outcomes.

Two online databases were searched (OVID MEDLINE and Embase) for articles using the following search terms: ((RDN OR renal sympathetic denervation OR catheter-based RDN OR kidney denervation OR renal denervation OR renal artery denervation) AND (atrial fibrillation OR AF)).

The inclusion criteria were: (1) randomised controlled trials, (2) published in English language, and (3) reporting on the recurrence rate of AF in patients with primary HTN following PVI ± RDN.

The exclusion criteria included: non-English language studies, non-peer-reviewed studies, observational and cohort studies, case series and reports, animal studies, abstract-only manuscripts, editorial comments, and opinion pieces.

**Figure 1:**
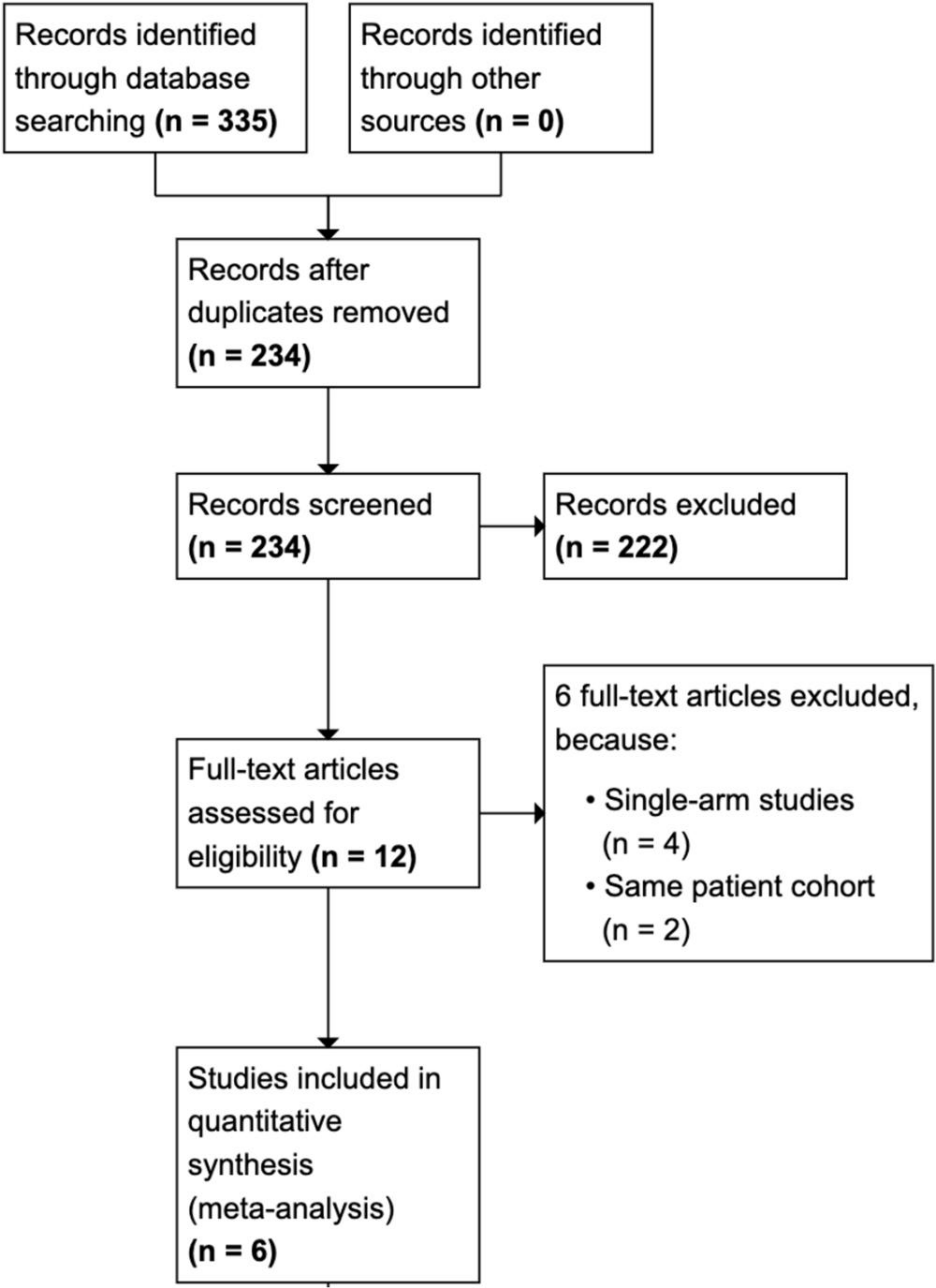
PRISMA flow diagram.

A PRISMA flowchart of the literature screen can be found in *Figure 1*.^8^

### Quality assessment of included studies

The Cochrane Risk of Bias (RoB 1.0) tool for randomised trials and a funnel plot were used to assess bias.^9,10^ In regard to the funnel plot, the risk ratio (RR) of the studies and the 95% confidence interval (CI) limits were plotted against their standard errors. Funnel plots were visually assessed for evidence of small study bias.

### Data extraction

Data extraction was done using a standardised form that included the primary author and year of publication, study design, sample size, age, gender, follow-up period, baseline characteristics of study participants, AF recurrence and follow-up blood pressures, and complications. Study authors were contacted for additional information on trial data and results when these were not available on the original publication.

### Data analysis

RevMan version 5.3 software (Cochrane Collaboration) was used for the pooled analysis. Inverse-variance random effects models were used for both dichotomous and continuous data as characteristics like age and gender differed across studies. RRs were used to compare effects for dichotomous data, whereas mean difference (MD) and standardised mean difference (SMD) were used to compare continuous data. Where SMD was used, it is important to note that the RevMan version 5.3 software implements the Hedges’ adjusted *g* formulation, which is very similar to Cohen's *d*, but includes an adjustment for small sample bias. The SMD represents the difference between the means of a variable, expressed not in its original unit, but in the unit of standard deviation. SMDs of 0.2, 0.5, and 0.8 are usually interpreted as associated with small, medium, and large effect sizes, respectively.

Regarding continuous data, if studies failed to report standard deviations (SDs), these values were estimated using the confidence intervals for the means reported.^11^

To further investigate for heterogeneity across studies, inconsistency index (*I*^2^) tests were used. An *I*^2^ value above 50% and *P*-value ≤ 0.10 from the χ^2^ test was considered to indicate potential heterogeneity.^12^ Where this was the case, sensitivity analysis was performed by removing the study deemed to have the highest risk of bias. If this was not possible or if considerable heterogeneity remained, then each study was removed in turn, and the effect on the values was observed.

## Results

### Study characteristics

There was a total of 637 patients across 8 randomised control trials (RCTs), of which 6 were multicentre, with 329 patients in the PVI + RDN group and 308 patients in the PVI alone group. Criteria for definition of resistant hypertension differed across studies (i.e. different BP cut-off levels and different number and type of used anti-hypertensive agents). The baseline characteristics of the included studies are summarised in *Table 1*.^13–18^

(A) Efficacy Outcomes

**Table 1:** Baseline characteristics of included studies

Primary author + Year	Kirstein (2022)	Turagam-HFIB 2 (2021)	Turagam-HFIB 1 (2021)	Steinberg (2019)	Kiuchi (2018)	Kiuchi (2016)	Pokushalov (2014)^a^
Study design	RCT	RCT	RCT	RCT	RCT	RCT	RCT
Study groups	PVI + RDN vs. PVI	PVI + RDN vs. PVI	PVI + RDN vs. PVI	PVI + RDN vs. PVI	PVI + RDN vs. PVI+ Spironolactone	PVI + RDN vs. PVI	PVI + RDN vs. PVI
Sample size (PVI + RDN/control)	39/22	28/22	13/17	154/148	33/36	21/24	41/39
Inclusion criteria	Refractory paroxysmal or persistent AF Drug-resistant HTN (daytime ambulatory SBP > 135 mmHg) At least three anti-hypertensive (including one diuretic) eGFR > 45	Paroxysmal or persistent AF Drug-resistant HTN (Office SBP ≥ 160 or DBP ≥ 100) At least one anti-hypertensive eGFR > 45	Paroxysmal or persistent AF Drug-resistant HTN (Office SBP ≥ 160 or DBP ≥ 100) At least one anti-hypertensive eGFR > 45	Paroxysmal AF Drug-resistant HTN (Office SBP ≥ 130 or DBP ≥ 80) At least one anti-hypertensive	Paroxysmal AF or symptomatic refractory AF Drug-resistant HTN (ambulatory SBP ≥ 130, ambulatory DBP ≥ 80) At least three anti-hypertensive eGFR ≥60 and microalbuminuria	Refractory paroxysmal or persistent AF Mostly drug-resistant HTN (130 > ambulatory SBP ≥ 100): mean of 3.2 anti-hypertensive agents, 2/3 with diuretic 30 ≤ eGFR ≤ 89 (or if eGFR > 60 and microalbuminuria)	Refractory paroxysmal or persistent AF Moderate drug-resistant HTN (Office BP ≥ 140/90) or severe drug-resistant HTN (Office BP ≥ 160/100) At least three anti-hypertensive (including one diuretic) eGFR ≥ 45
% PAF vs. % persistent AF	50.8/49.2	70.0/30.0	66.7/33.3	100/0	100/0	60.0/40.0	43.8/56.2
Age (Mean ± SD) (PVI + RDN/control)	66.3 ± 7.90/63.0 ± 9.90	64.0 ± 7.00/65.0 ± 8.00	59.0 ± 10.0/68.0 ± 9.00	59 (54–65)^b^/60 (58–65)^b^	56.8 ± 6.50/58.4 ± 5.10	68.0 ± 9.0/66.0 ± 9.0	56.0 ± 6.00/56.0 ± 6.00
Female (%) (PVI + RDN/control)	48.7/45.5	42.9/36.4	38.5/47.1	40.9/38.5	24.2/16.7	38.1/33.3	24.4/38.5
Baseline SBP (mmHg) (PVI + RDN/control)	162.0 ± 18.60/167.1 ± 18.50	146.6 ± 20.6/143.4 ± 18.4	147.0 ± 31.0/153.0 ± 20.0	150.0 ± 9.50/151.0 ± 9.31	142.0 ± 6.00/140.0 ± 6.00	Drug-controlled HTN	163.0 ± 18.0/164.0 ± 17.0
Baseline DBP (mmHg) (PVI + RDN/control)	87.5 ± 14.1/91.4 ± 10.6	81.4 ± 13.4/79.1 ± 12.4	84.1 ± 25.0/88.0 ± 12.0	90.0 ± 6.33/90.0 ± 9.31	103.0 ± 8.00/103.0 ± 7.00	Drug-controlled HTN	89.0 ± 11.0/88.0 ± 11.0
Baseline eGFR	77.6 ± 17.4/72.7 ± 14.2	NR	NR	79.0 ± 11.0/76.0 ± 11.0	69.2 ± 6.70/66.7 ± 7.70	59.3 ± 13.3/60.5 ± 15.9	75.5 ± 9.20/77.0 ± 8.50
PVI method	Radiofrequency ablation	Radiofrequency ablation	Cryoballoon catheter	Radiofrequency ablation	Radiofrequency ablation	Radiofrequency ablation	Radiofrequency ablation
RDN method	EnligHTN	Vessix	ThermoCool	Irrigated tip and RDN catheter	EnligHTN	Irrigated tip	ThermoCool (*n* = 20), Simplicity (*n* = 21)
Follow-up (months)	24	24	24	12	12	12	12

RCT, randomised controlled trial; PVI, pulmonary vein isolation; RDN, renal denervation; DBP, diastolic blood pressure; SBP, Systolic blood pressure; eGFR, estimated glomerular filtration rate; HTN, hypertension; AF, atrial fibrillation; BP, blood pressure; SD, standard deviation.

^a^Pokushalov (2014) reported grouped results from two different RCTs.

^b^This study reported data as median and interquartile range.

### Atrial fibrillation recurrence

**Figure 2:**
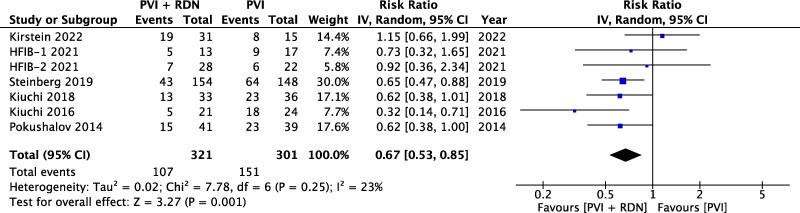
Effect of renal denervation on atrial fibrillation recurrence.

The pooled analysis showed that AF recurrence was significantly lower in the PVI + RDN (33.3%) group in contrast to the PVI (50.2%) group [RR = 0.67 (0.53, 0.85), *P* = 0.001, *I*^2^ = 23%] (*Figure 2*). The number needed to treat (NNT) to prevent recurrence was 5.9 patients.^19^

### Blood pressure

All included trials reported follow-up data on blood pressure. One of the trials included patients with drug-controlled HTN and thus was excluded from the meta-analysis.^14^ Kiuchi (2018) reported blood pressure as ambulatory as opposed to in-office.^15^ To account for the difference in measurements of the outcome, SMDs were used.^10^ Twelve-month follow-up data was used here.

**Figure 3:**
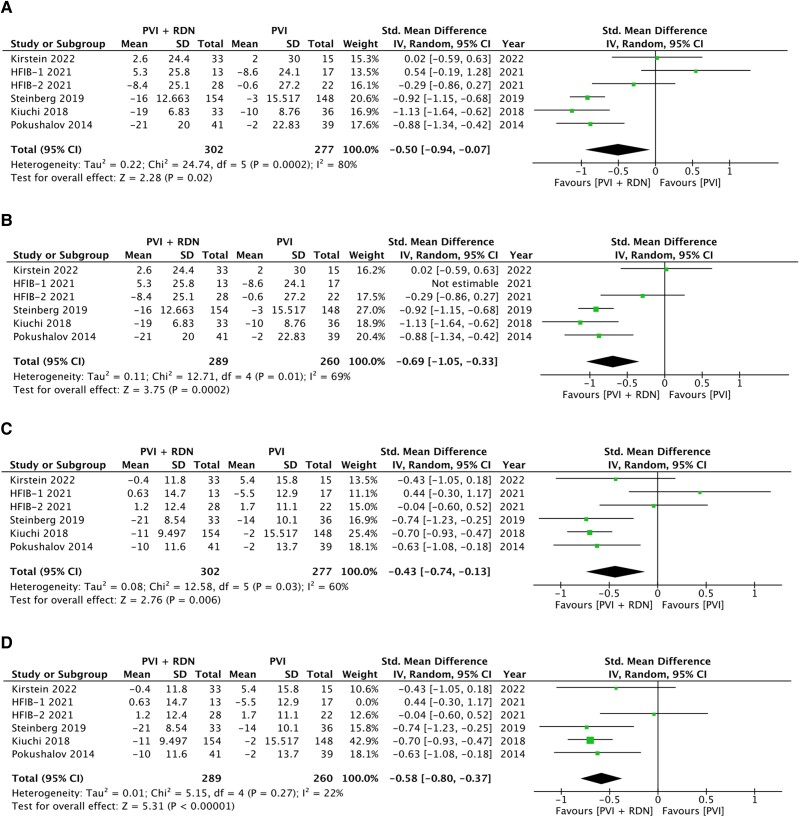
Effect of renal denervation on blood pressure. Forest plots of standardised mean difference for (*A*) systolic blood pressure, (*B*) systolic blood pressure sensitivity analysis after HFIB-1 removal, (*C*) diastolic blood pressure, and (*D*) diastolic blood pressure sensitivity analysis after HFIB-1 removal.

The pooled results showed a significant decrease in systolic blood pressure (SBP), with medium effect size, as reflected by an SMD of 0.5 in the PVI + RDN group compared with the PVI alone group (*P* < 0.05). Similarly, diastolic blood pressure (DBP) was reduced by an SMD of 0.43 (*P* = 0.006) (*Figure 3A* and *C*). There was a high heterogeneity in both the SBP and DBP pooled analyses with *I*^2^ values of 80 and 60%, respectively, and χ^2^ test *P*-values ≤ 0.10.

**Figure 4:**
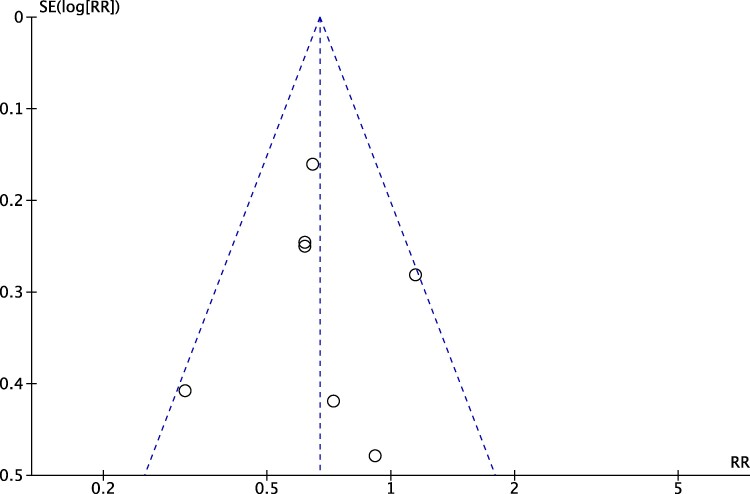
Funnel plot analysis.

Sensitivity analysis was conducted through the removal of the HFIB-1 trial (*Figure 3B* and *D*). This trial was removed to try and adjust for publication bias (*Figure 4*). The results indicated a significant difference of *P* = 0.0002 for SBP and a significant difference of *P* < 0.00001 for DBP. Though the SBP and DBP pooled analyses heterogeneity decreased to *I*^2^ of 69 and 22% respectively, considerable heterogeneity remained in the pooled SBP data (*Figure 3B* and *D*).

(B) Safety Outcomes

### Estimated glomerular filtration rate

**Figure 5:**
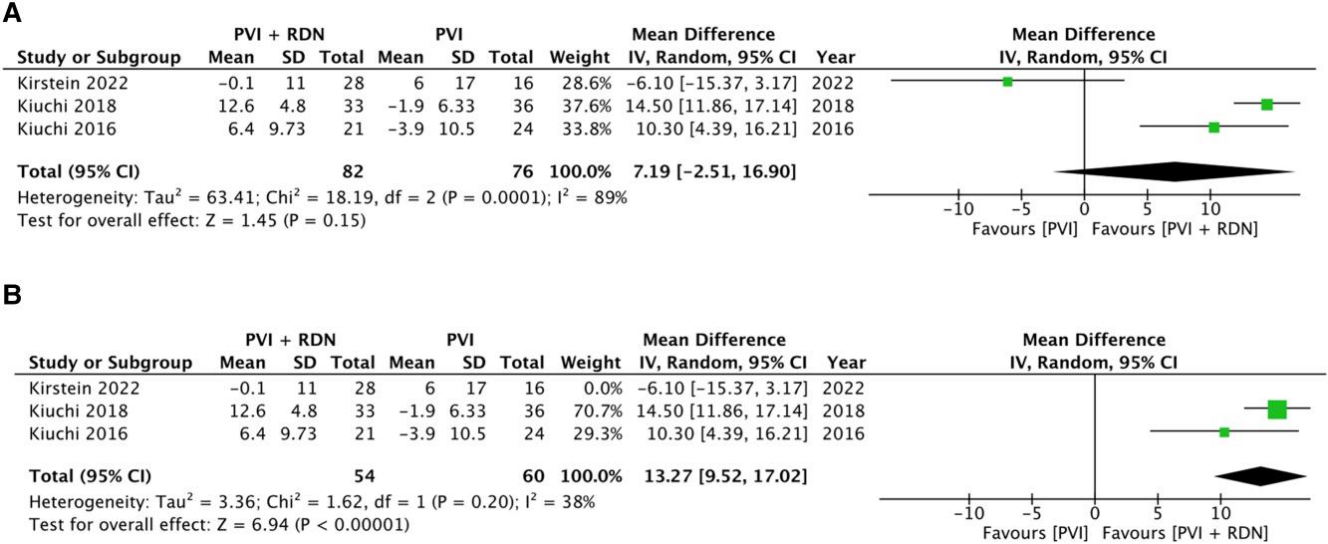
Effect of renal denervation on estimated glomerular filtration rate. Forest plots of mean difference for (*A*) estimated glomerular filtration rate and (*B*) estimated glomerular filtration rate sensitivity analysis (Kirstein 2022 not included in pooled analysis).

Baseline and follow-up eGFR data for both PVI + RDN and PVI groups were reported in three trials.^14,15,18^ The pooled analysis was conducted using the most recent follow-up data. There was an MD in eGFR of +7.19 mL/min/1.73 m^2^ in the PVI + RDN group compared with the PVI alone group, but the difference was not significant (*P* = 0.15) (*Figure 5A*). Owing to the large heterogeneity (*I*^2^ = 89%), a sensitivity analysis was conducted. Following the removal of Kirstein (2022), the results indicated a significant difference (*P* < 0.00001) and reduced heterogeneity (*I*^2^ = 38%) (*Figure 5B*). This effect was not observed following the removal of any of the other papers.

### Procedural complications

Five trials reported data on peri- and post-procedural complications in both groups.^15–18^ Kiuchi (2016) reported complications only in the PVI + RDN group and Pokushalov (2014) failed to specify the group in which the single complication (cardiac tamponade following PVI) occurred.^13,14^

Steinberg (2019) and Pokushalov (2014) attributed all complications to the PVI procedure.^13,16^ Procedural complications specifically related to RDN were reported in the HFIB-1 and Kirstein (2022) trials. Renal artery stenosis in the PVI + RDN group was reported in 3/13 (23.1%) patients in the HFIB-1 trial and 1/39 (2.56%) patients in the Kirstein’s (2022) study.^17,18^ Participant recruitment for the HFIB-1 trial was terminated early due to complications.^17^

Kirstein (2022) reported 12 non-fatal, peri-procedural complications in 9 patients across both groups (4/39 in the RDN + PVI group and 5/22 in the PVI group), but failed to specify what these events were.^18^ Steinberg (2019) reported eight non-fatal major adverse cardiac events in each group.^16^ Despite specifying what these events were, it failed to report how many patients experienced each event (*Table 2*).

**Table 2:** Peri- and post-procedural complications

Primary author + Year	Kirstein (2022)		Turagam-HFIB 2 (2021)		Turagam-HFIB 1 (2021)		Steinberg (2019)		Kiuchi (2018)		Kiuchi (2016)		Pokushalov (2014)	
Study groups	PVI + RDN	PVI	PVI + RDN	PVI	PVI + RDN	PVI	PVI + RDN	PVI	PVI + RDN	PVI	PVI + RDN	PVI	PVI + RDN	PVI
Renal artery stenosis	1/39	0	0	0	3/13	0	0	0	0	0	0	NR	0	0
Stroke	0	0	0	0	0	1/17	?	?	0	0	0	NR	0	0
Systemic embolism	0	0	0	0	0	0	?	?	0	0	0	NR	0	0
Vascular/access-related complications e.g. femoral fistula, pseudoaneurysm, haematoma	2/39	0	2/28	0	0	1/17	6/154	4/148	0	0	0	NR	0	0
Cardiac tamponade	0	2/22	0	0	0	0	0	1/148	0	0	0	NR	?	?
Procedural death	0	0	0	0	0	0	0	0	0	0	0	NR	0	0
Death during follow up	0	0	0	0	0	0	2/154	2/148	0	0	0	NR	0	0
Transient phrenic nerve palsy	?	?	0	0	0	0	1/154	1/148	0	0	0	NR	0	0
Pneumothorax	?	?	0	0	0	0	0	1/148	0	0	0	NR	0	0
Renal artery dissection in procedure	0	0	0	0	3/13	0	0	0	0	0	0	NR	0	0

PVI, pulmonary vein isolation; RDN, renal denervation; NR, not reported.

### Risk of bias

**Figure 6:**
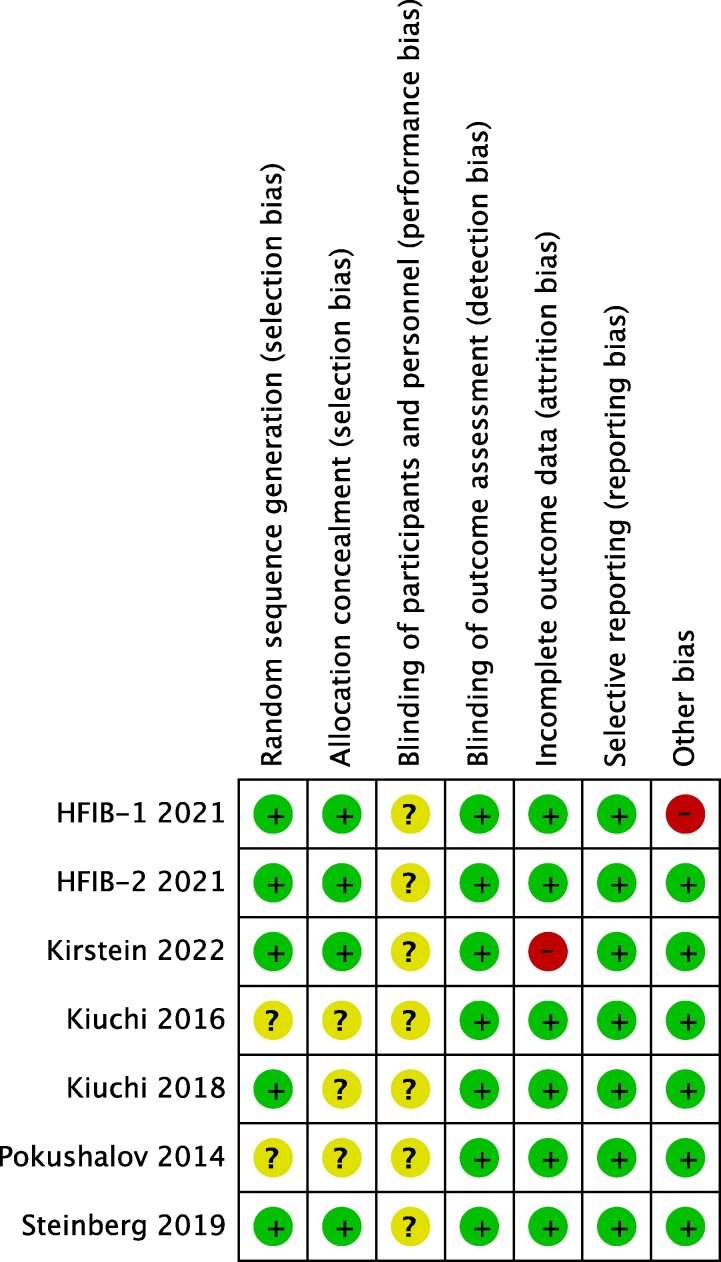
Cochrane RoB for randomized trials

In certain studies, the method of random sequence generation and allocation concealment was not clearly defined.^13–15^ There was the potential for performance bias in all the studies as the same operators performed both PVI and RDN, thus they were not blinded to the groups. Attrition bias was low for all studies except Kirstein (2022), with very low numbers lost to follow-up. In the Kirstein (2022) trial, there were 11 withdrawals and 5 missed visits during the follow-up period and no evidence of an intention-to-treat analysis being carried out.^18^ HFIB-1 was terminated prematurely due to a large number of renovascular complications.^17^ Therefore, pooled outcomes that included this study should be assessed with caution. All studies were unclear bias in at least one risk of bias domain (*Figure 6*).

The funnel plot for AF recurrence results show that study RRs are symmetrical around the overall RR and lie within or on the 95% CI limits, indicating a low risk of publication bias (*Figure 4*).

## Discussion

This meta-analysis examined the findings from eight trials investigating the effects of RDN as an adjunct to PVI in the treatment of AF in patients with drug-resistant HTN. The pooled results showed that RDN alongside PVI significantly reduced 12-month AF recurrence, 12-month SBP and DBP.

HTN is the main risk factor for AF and currently the mainstay of treatment is a combination of lifestyle modification and drug therapy.^20^ HTN is believed to have a neurogenic component, where the kidneys play an important role. It has been shown that the activation of efferent sympathetic renal nerve fibres results in: (i) renin release via β1 adrenoreceptor activation in the juxtaglomerular cells, with downstream activation of the renin–angiotensin–aldosterone system, (ii) increase in renal tubular sodium reabsorption via α adrenoceptors, and (iii) decrease in renal blood flow.^21^ Ultimately, this leads to sodium and water retention, and increased systemic vascular resistance, which induces left atrial fibrosis and conduction block in the left atrium, and may potentially result in AF development.^22^

Numerous studies have looked into the use of RDN in HTN treatment.^23^ Blood pressure lowering effects are thought to be achieved by the ablation of renal sympathetic nerve fibres within the adventitial layer of the renal arteries. Consequently, reducing sensory input from the kidneys to the cerebral hypothalamic centres and modulating sympathetic outflow to kidneys, heart, and peripheral blood vessels.^24^ Ultimately, this reduces cardiac stimulation and leads to reduced cardiac fibrosis, hypertrophy, and arrhythmogenicity.^25^ Catheter ablation through PVI has been an effective modality in reducing recurrence of AF especially in patients with resistant AF to medical therapy.^26^ By reducing sympathetic activity, RDN may have a concurrent anti-arrhythmic feature which might further reduce recurrence of AF post-catheter ablation.

Several clinical studies investigating RDN and PVI compared with PVI alone are ongoing. One of these trials is the prospective, randomised, controlled, multicentre ASAF trial (ClinicalTrials.gov Identifier: NCT02115100). It aims to explore the time to first detection of AF > 30 s, in patients with paroxysmal or persistent AF with uncontrolled HTN or signs of sympathetic overdrive. Recruitment to this trial has been completed and the results would be of high impact. Another ongoing prospective, controlled, and randomised trial is the Ultrasound-Based Renal Sympathetic Denervation as Adjunctive Upstream Therapy During Atrial Fibrillation Ablation (ULTRA-HFIB) (ClinicalTrials.gov Identifier: NCT04182620) which aims to recruit 160 patients undergoing AF ablation procedure. This trial aims to determine the role of RDN plus PVI in the prevention of AF recurrence and aims to be completed by December 2023.

### Clinical implications

RDN in adjunct to PVI has been shown to reduce AF recurrence and blood pressure and thus should be considered for the treatment of patients with AF and drug-resistant HTN. Though some studies reported peri-procedural complications arising from the RDN process (e.g. renal artery stenosis), differences in reporting across studies did not allow us to performed a pooled analysis. Importantly, the three trials reporting on eGFR suggested no harm or deterioration occurring as a result of RDN.

### Limitations

The main limitation of this meta-analysis is likely biases arising from individual included studies. Several measures were taken to mitigate the effect of biases including: (1) not including studies with duplicated patient populations; (2) funnel plot analysis to probe for publication bias; and (3) ensuring consistency in inclusion criteria. Despite this, other forms of bias such as language bias, arising from only including English language studies were still likely present. Another limitation is the inclusion of small, potentially underpowered studies. Yet, incorporating these smaller studies is important since they are likely to reflect any heterogeneity that might manifest in clinical settings.^27^

All systematic reviews published on this topic, except for one, include duplicated patient populations by incorporating data from at least two trials by Pokushalov and colleagues (2012, 2014) and Romanov (2017).^13,28,29^ Our review includes only Pokushalov (2014), as it encompasses also the participants from the 2012 trial. Romanov (2017) was excluded as it was a sub-analysis of patients from the two trials published by Pokushalov and colleagues with data from implantable cardiac monitors. This study stands as the first meta-analysis on this topic to exclusively include RCTs, to ensure that only higher quality evidence was utilised. It also incorporates the most recent data from the Kirstein (2022) trial. Notably, this is the only trial to date to conclude that RDN did not improve AF outcomes and is one of only two trials to report peri-procedural complications of RDN, thereby somewhat mitigating outcome-reporting bias.^18^

None of the trials used invasive sham controls, considered by many to be a requirement for trials investigating device-based HTN therapies.^30^ However, there is also evidence to suggest that the use of sham controls is no more effective than extensive use of 24-h ambulatory SBP.^31^

## Conclusion

This meta-analysis revealed that RDN alongside PVI significantly reduced 12-month AF recurrence, 12-month SBP and DBP. It would be valuable to expand the consideration of RDN as a treatment option for AF, in addition to PVI in patients with drug-resistant HTN. Given the potential benefits of RDN, additional trials in this area would be beneficial to further clarify the efficacy and safety of this approach in the AF population.

## Data Availability

All data used in this article were retrieved from the eight included randomized controlled trials, and are available in the published versions of the manuscripts.

## Lead author biography

Karish Thavabalan is a final year MBBS student at UCL having graduated from Imperial College Business School with a 1st Class degree in Management. Passionate about advancing cardiovascular care, his expertise lies in the intersection of renal denervation and atrial fibrillation treatment. With a commitment to evidence-based medicine, his work aims to contribute valuable insights to the evolving field, fostering innovation and enhancing therapeutic strategies for individuals battling atrial fibrillation.
